# 

**Published:** 1911-06-10

**Authors:** 


### VACCINATION ACTS (REPEAL).

A Bill has been introduced in the House of Commons by Mr. Black to repeal the Vaccination Acts; it is supported by Sir George White, Mr. James Parker, Mr. M. M'Callum, and Mr. Kellaway. It forbids the payment out of the poor rates or any other public fund for the performance of vaccination or for the supply of vaccine lymph, and provides that it shall not be lawful to require that vaccination shall be a condition of admission to the Services or be ground for disqualification from any public employment.

### APPOINTMENT OF MEDICAL OFFICERS OF HEALTH.

Sir Charles Henry asked the President of the Local Government Board, if he would instruct that, in the re-adjustment of districts in counties for sanitary supervision, those whole-time Medical Officers of Health who previously to these arrangements held appointments in these districts, should receive consideration in the selection for medical officers for the newly-formed districts? In reply Mr. Burns explained that the selection of the medical officer of health rests with the local authority making the appointment, but he said he entirely agreed with the view that, in making fresh appointments, the claims of any efficient officers who may be displaced in consequence of the new arrangements should receive full consideration.

### BUILDINGS CONTEMPLATED AND IN PROGRESS.

Aylesbury.—Mr. F. Taylor, A.R.I.B.A., of Aylesbury, on receiving £5 deposit, will send tender forms and quantities for the proposed alterations to the infirmary.

Birkenhead.—Additions are contemplated to the Workhouse Nurses' Home at a cost of close on £2,000. It is also proposed to convert the probationary lodge into an isolation block at a cost of £1,025.

Bradford.—Mr. Holland, architect to the board, has prepared plans for extensions and additions to the medical officer's residence. At the workhouse, quantities may be had on deposit of 10s. 6d.

Barrow-upon-Soar.—Certain alterations and additions are projected to the Union Workhouse. Quantities may be obtained from the clerk to the Guardians, Mount Sorrel, Loughborough.

Billericay.—Mr. Hugo Bird, of Brentwood, being appointed architect for the erection of nurse's homes in connection with the Billericay Workhouse, will send particulars to builders desiring to tender on deposit of £1 1s.

Littlehampton.—The building works are in full swing for the Littlehampton New Hospital. The total cost will be about £2,100, and the building will contain six-bed wards for men and women respectively, isolation ward, operating room, anæsthetic room, and staff accommodation.

Malvern.—A well-equipped and up-to-date hospital has been erected and formally opened. Mr. William Henman, F.R.I.B.A., is the architect, and the contractors are Messrs. J. and A. Brazier.

Newton Abbot.—Mr. S. Segar, F.I.A.S., of Newton Abbot, is carrying out a new laundry at Brookhill, Highweek, for the Guardians of the Union. The tenders have just been received.

Neath.—The Guardians have resolved to make application to the Local Government Board for sanction to borrow £48,500 for the erection of a new infirmary. Accommodation will be provided for close on 500 persons.

Oxford.—At a meeting of the Governors' Court of the Radcliffe Infirmary, the plans prepared by Mr. Edward Warren, architect, consulting with Dr. Mackintosh, of Glasgow, for new buildings were considered, and it was decided to instruct the Building Committee to invite tenders. The approximate cost is about £25,000.

Pontypridd.—The local Board of Guardians propose to erect an Infirmary and Nurses' Home. Messrs. Thomas and Morgan, architects, have been instructed to prepare a design.

Paddington (North).—An addition is proposed for the Children's Hospital, Paddington Green. Mr. A. E. Munby has obtained a consent under the London Building Acts.

Paddington.—The Guardians have instructed their architect to prepare sketch plans and approximate estimate for an addition to the Workhouse dining hall, to accommodate 300 women. Mr. E. Howley Sim is the architect to the Guardians.

Portsmouth.—A tender of £290 has been accepted for the erection of a Tuberculin Dispensary for the Corporation of Portsmouth.

Rotherham.—The Hospital Committee invite tenders for the erection of a Smallpox Hospital, comprising administration, laundry and ward blocks, boundary walls, construction of roadways, and laying-out of grounds. Tenders are to be delivered before June 14, and plans may be inspected at the Town Hall.

Sedgefield.—Alterations and additions are presently to be commenced at the Isolation Hospital. Form of tender may be had on deposit of 21s. from Mr. James Stones, surveyor to the Sedgefield Council.

Wandsworth.—The Guardians invite tenders for additions and renovations at the St. John's Hill Infirmary, which are to be returned before June 15.

York.—The drawings, etc., for the proposed Hospital for open-air treatment may be seen at the City Engineer's office in the Guildhall, York.

### APPOINTMENTS VACANT.

Full particulars of these vacancies can be obtained on reference to our advertisement columns:

Beckett Hospital, Barnsley.—Second House Surgeon.

Coventry and Warwickshire Hospital.—Junior House Surgeon.

Royal Albert Hospital, Devonport.—Assistant House Surgeon.

Kent and Canterbury Hospital.—House Physician.

Royal Surrey County Hospital, Guildford.—Assistant House Surgeon.

The Prince of Wales's General Hospital, Tottenham, N.—Honorary Surgeon.

Bradford Royal Infirmary.—Resident Surgical Officer.

Birmingham and Midland Eye Hospital.—Junior House Surgeon.

Evelina Hospital for Children, Southwark.—House Physician.

Hospital for Sick Children, Great Ormond Street.—House Surgeon and Ophthalmic Surgeon.

### JOTTINGS.

At a recent meeting of the General Committee of the West Ham and Eastern General Hospital, Mr. Walter Smith, a member of the General Committee of Management of the hospital, mentioned that at one of the West Ham schools a boy of eight, who contributed 4s. 4d. to the West Ham Hospital School Children's Fund, when questioned about the manner of collecting the sum, replied that it represented one penny per week which his mother had promised to give him if he went without sugar in his tea.

Our contemporary, *Mercy and Truth*, gives an account of the opening of the Seth Hiranand Hospital, Shikarpur, Sindh, from which we gather that the hospital owes its origin to the generosity of Seth Hiranand, a Hindu banker, who expressed his willingness to build the institution provided that the doctors of the C.M.S. would make an annual visit to the town. Satisfactory terms having been arranged the building was erected and opened with due ceremony on February 28 of this year. The institution contains a consulting-room, a special room for women, a well-appointed operating theatre, another theatre for septic cases, a dressing-room and a dark room for ophthalmic examinations. At present there is no accommodation available for in-patients, a fact which handicaps the work of the surgical staff.

### COMING EVENTS OF THE WEEK.

[Communications for this column must be sent to 29 Southampton Street, Strand, before noon on Wednesday.]

Monday, June 12.—West London Hospital, Hammersmith Road. Demonstration by Surgical Registrar, 10 a.m. Pathological demonstration, 12 noon. Lecture, "Rectal Surgery," by Mr. Edwards, 5 p.m.

Polyclinic, 22 Chenies Street. Dr. Adamson, 4 p.m., Skin Clinique. Dr. Macnamara, "Psychoneuroses," 5.15 p.m.

Tuesday, June 13.—Royal Hospital for Incurables, Putney. Countess of Dalhousie opens Sale of Work, 2.30 p.m.

National Hospital, Queen's Square, W.C. Lecture, "Significance of Tremor," Dr. R. Russell, 3.30 p.m.

Kensington and Fulham General Hospital. Coronation Festival Dinner, Trocadero, 8 p.m. Lord Claud Hamilton's reception precedes the dinner at 7.30 p.m.

West London Hospital, Hammersmith Road. Lecture, "The After Treatment in Gynæcological Operations," Dr. Simson, 5 p.m.

Central London Throat Hospital. Mr. Nourse, Accessory Services, 3.45 p.m.

Wednesday, June 14.—West London Hospital, Hammersmith Road. Practical Medicine, Lecture IV., by Dr. Beddard, 5 p.m.

British Home and Hospital for Incurables. Jubilee Festival Dinner, Princes Restaurant, Piccadilly, F. A. Beven, D.L., J.P., presiding, 7 p.m.

Polyclinic, 22 Chenies Street. Mr. Spencer, "Inflammatory Conditions of the Mouth," 5.15 p.m.

Friday, June 16.—National Hospital, Queen's Square, W.C. Lecture, "Spinal Injuries," by Mr. P. Sargeant, 3.30 p.m.

London Homœopathic Hospital, Great Ormond Street. Lecture, "Tubercular Lymphadenitis," by James Eadie, M.B., F.R.C.S., 5 p.m.

